# Dietary Polyphenols—Important Non-Nutrients in the Prevention of Chronic Noncommunicable Diseases. A Systematic Review

**DOI:** 10.3390/nu11051039

**Published:** 2019-05-09

**Authors:** Wojciech Koch

**Affiliations:** Chair and Department of Food and Nutrition, Faculty of Pharmacy, Medical University of Lublin, 4a Chodźki Str., 20-093 Lublin, Poland; kochw@interia.pl

**Keywords:** chronic noncommunicable diseases, polyphenols, flavonoids, dietary intake

## Abstract

The improvement of the social and economic conditions of society has eliminated the threat of death from the majority of infectious diseases. However, the rapid progress of civilization has created new possibilities for the appearance of factors with adverse effects for the health of society. This has led to increased morbidity from certain diseases, the presence of which had not been observed several centuries ago. Chronic noncommunicable diseases (e.g., cancers, cardio-vascular disorders, diabetes, obesity, neurodegenerative diseases) result from an inappropriate relationship between people and their environment. The common characteristic for all chronic diseases is a “new” form of inflammation, very often called metaflammation, which is considered as a subclinical, permanent inflammation. As a result, metabolic cascade, including cellular oxidative stress, atherosclerotic process, and insulin resistance, occurs, which slowly generates significant deterioration in the organism. Polyphenols are the major group of non-nutrients, considering their diversity, food occurrence, and biological properties. The current review aims to present a wide spectrum of literature data, including the molecular mechanism of their activity and experimental model used, and summarize the recent findings on the multitude of physiological effects of dietary polyphenols towards the prevention of several chronic diseases. However, despite several studies, the estimation of their dietary intake is troublesome and inconclusive, which will be also discussed.

## Highlights

During recent decades; a significant increase in chronic noncommunicable diseases has been observed; Polyphenols are the main group of biologically active non-nutrients present in food; Estimation of dietary intake of polyphenols is difficult; Beneficial effects of phenolics towards the prevention of lifestyle diseases are reviewed

## 1. Introduction

The rapid progress of humanity observed starting from the Industrial Revolution and especially after World War II has led to an increase in the length and quality of life. Rapid development of medical and pharmaceutical sciences has led to an improvement in diagnosis and the introduction of new drugs (e.g., vaccination or antibiotics). Together with the improvement of the social and economic conditions of society, this has eliminated the threat of death from the majority of infectious diseases [1,2]. However, the rapid progress of civilization has created new possibilities for the appearance of factors with adverse effects for the health of society. The Western diet, with its high saturated fat and sugar intake, physical inactivity, insufficient sleep, strong psychological stress, low sun exposure, environmental pollution, and smoking or alcohol abuse, are some of the most important factors which have been discussed during the last 20 years in numerous papers and reports, leading to the description of the many so-called “diseases of civilization” [1,3,4].

## 2. Chronic Noncommunicable Diseases

Lifestyle diseases are classified as diseases resulting from an inappropriate relationship between people and their environment and lifestyle [2]. They are often described as chronic noncommunicable diseases (NCDs) and are, in general, treated as the same group of diseases. The list of impairments of such a type is increasing. Recently, Egger and Dixon [5] classified chronic diseases with lifestyle or environmental determinants into ten categories:

- cardio- and cerebrovascular diseases
- cancers with lifestyle components
- endocrine/metabolic disorders
- gastrointestinal disorders
- kidney disease
- mental/central nervous system (CNS) health
- musculoskeletal disorders
- respiratory diseases
- reproductive disorders
- dermatological disorders

### 2.1. Historical Background

On the basis of anthropological and medical reports, several authors suggest that these diseases are not observed or are rare in hunter-gatherer or nonwesternized populations [6,7,8]. This hypothesis is supported by historical records of explorers and adventurers which support the evidence of the superior health of pre-agriculture traditional populations [6]. Deterioration of diet did not start in the last 50 or 100 years; rather, it started much earlier. The Agricultural Revolution started about 11,000 years ago in the Middle East and quickly spread around the globe [3,9,10]. “Industrial” food production significantly altered the human diet and lifestyle, which was associated with the introduction of cereal grains as staple foods, domesticated meats, nonhuman milk, legumes, and other cultivated plant foods and later a common use of sucrose and alcoholic drinks [3,9]. According to Carrera-Bastos et al. [1], the superior physical health and body composition of hunter-gatherers and other traditional populations minimally affected by modern diet and lifestyle were not primarily due to genetics, but to environmental factors. They also suggest that during the last several thousand years, no significant gene changes have occurred to protect humans from chronic diseases caused by modern habits. Based on the anatomical, biomechanical, and isotope analysis of the hominin skeletons, several typical diet and lifestyle characteristics of the hunter-gatherers and traditional populations may be observed; these are presented in Table 1.

The 20th century brought an improvement in sanitation, hygiene, and immunization and the development of antibiotics, which resulted in the overcoming of “germ diseases” [24]. In the 1970s and 1980s in the developed countries of North America, Europe, and the Asian-Pacific region, the process of the “epidemiological transition” started, when chronic diseases began to be more frequent compared to infectious. Currently, this can be observed in developing countries such as Brazil, China, Russia, or India [5,25]. According to Egger and Dixon, the progress of civilization tamed infectious diseases, but surprisingly instigated an unhealthy style of life, which resulted in the rapid outbreak of NCDs [5].

### 2.2. Current Findings on NCDs

The background of NCDs is rather complicated, in contrast to infectious diseases which are always connected with germs. The common characteristic for all chronic diseases is a “new” form of inflammation, which from the beginning of the 1990s has been called metaflammation [5]. This term is used to describe a form of low-grade, chronic, and systemic inflammation, originally associated with obesity. Recent findings have revealed that “metaflammation” is not limited to obesity, but also related to other lifestyle diseases and conditions, for example heart disease, type 2 diabetes, many forms of cancer, osteoporosis, and even central nervous system diseases (depression or dementia) [26,27,28,29]. Metaflammation is considered a part of a metabolic cascade, including cellular oxidative stress, atherosclerotic process, and insulin resistance. As a result, dysmetabolism, induced allostatic overload, and finally chronic impairment can be observed [30,31]. Factors which induce metaflammation have largely arisen since the industrial revolution and have been named “anthropogens” or man-made environmental inducers [32,33,34].

Anthropogens induce low, subclinical, but persistent immune-response to a nonlife-threating situation. This situation could become dysmetabolic if exposure starts to be prolonged [5]. Moreover, low-grade chronic inflammation is often associated with man-made environmental inducers; for example, chronic psychological stress, improper diet, environmental pollution, smoking, and drug and alcohol abuse [35,36,37,38,39]. Numerous studies have been performed indicating different factors regarding the progress of low-grade chronic inflammation and finally its contribution to NCDs. The most important are insufficient sleep (fewer than 6 h per 24-h day), chronic vitamin D deficiency, physical inactivity, and broadly understood nutritional changes [3,17,40,41,42]. Surprisingly, these are in contrast to the factors presented in Table 1, which were characteristic for hunter-gatherers and other traditional populations.

### 2.3. Nutritional Changes Associated with NCDs—Potential Role of Antioxidants

The important role of nutrition for the prevention of chronic diseases is already well known. Inadequate as well as overnutrition may lead to serious consequences [5,43]. Recent statistics have shown that improper nutrition accounts for up to two-thirds of the risk for type 2 diabetes, cardiovascular diseases (CVD) [44], and other chronic impairments [45]. The diets of industrialized societies are significantly changed compared to Paleolithic or even Ancient Egyptian or Medieval populations [1]. Chronic disease progress has been associated with important nutritional aberrations which have occurred during last few centuries, regarding specific nutrient intake [46], overall meal patterns, food processing, and general food product characteristics, including glycemic index and macro- and micronutrient density [1,47]. Numerous studies have indicated dietary risk factors associated with the increased risk of developing noncommunicable diseases:

- excessive energy intake from high energy-dense/low nutrient-dense products [5,48]
- common consumption of products with high glycemic load and index [49,50]
- low dietary fiber intake (in particular, in soluble form) [51,52]
- low dietary poly-unsaturated fatty acid (PUFA) intake [48,53]
- low dietary omega-3 fatty acid intake [53]
- high dietary trans fatty acid intake [54]
- high dietary sodium intake and improper sodium/potassium status [1,53]
- low intake of fruits and vegetables [53,54]
- hyperhomocysteinemia associated mostly with a high animal-protein diet [55]

During the last two decades, it has been proposed that oxidative stress, caused by reactive oxygen species (ROS), may be a key factor in the development of insulin resistance, diabetes, cardiovascular diseases, neurodegenerative disorders, and other NCDs [56,57,58]. Prolonged exposure of β-cells to oxidative agents (e.g., H_2_O_2_) results in their dysfunction and induced insulin resistance [59] and that the use of antioxidants improves insulin sensitivity, which has been proved in in vitro and animal model studies [60]. The role of ROS in the initiation, progression, and clinical consequences of cardiovascular diseases is already well known and has been described in several research papers proposing different mechanisms of antioxidant action in the prevention of CVD [61,62,63,64,65].

Although changes in lifestyle and diet (according to the characteristics presented above) are still considered crucial, many authors suggest that increased dietary intake of antioxidants is one of the most important factors in the prevention of NCDs, especially when those antioxidants are taken with a normal diet and not via dietary supplements [66,67,68,69]. The human diet contains many different antioxidants, including vitamins (C, E, β-carotene), trace elements (copper, iron, zinc, selenium), or plant metabolites (polyphenols, carotenoids) [70,71]. Although phytochemicals are often considered antinutrients or non-nutrients, during the last 15 years several findings have shed new light on their positive impact on the human organism, including their significant role in the prevention of chronic diseases [72,73]. Plant foods contain a wide spectrum of secondary metabolites among which polyphenols are one of the most abundant and nutritionally important phytochemicals [73]. There are over 8000 phenolic structures currently known and over 500 are present in plant foods and are considered dietary polyphenols. Polyphenols are synthetized from the combination of derivates formed from phenylalanine and acetic acid in two biochemical pathways—the shikimate and the acetate pathways. This is a very wide and complex group of phytochemicals, for which the common feature is the presence in their structure of at least one or more phenolic groups, which are responsible for the strong reducing properties of polyphenols. In nature, they occur as simple molecules, for example phenolic acids or flavonoids, or as very complicated polymerized macromolecules with molecular weights of greater than 30,000 Da, such as tannins. The diversity of polyphenols is increased, as they appear in the form of glycosides with one or two sugars, of which glucose is the most abundant molecule. Other sugar residues involve galactose, rhamnose, arabinose, xylose or glucuronic and galacturonic acids, and many others. Direct connections between sugar residues and aromatic carbon atoms (C–C binding) are also common.

## 3. Polyphenols—Estimation of Daily Dietary Intake Problems

Major classes of polyphenols involve simple phenols, phenolic acids, acetophenones, phenylacetic acids, coumarins, anthraquinones, xanthones, stilbenes, lignans, and flavonoids. The latter are the most complicated and the most widely distributed in plants. Flavonoids account for approximately two-thirds of dietary polyphenols and the remaining one-third are phenolic acids, being the second most important group of phenolics present in foods [74]. Flavonoids are an integral part of animal and human diets and plants are their only sources, as they cannot be synthesized by animals [75]. The most frequent class of flavonoids found in foods are flavonols, which are predominant in fruits in the form of different glycosides. In vegetables, the quercetin derivates are the most abundant flavonoid [76,77]. Exact estimation of daily dietary intake of flavonoids is difficult given their complicated distribution in plants, variety of classes, techniques used (analytical or calculating methods), and food habits. Moreover, several factors that influence their level in plants (germination, degree of ripeness, light), different flavonoid structures which still have not been identified and food processing cannot be ignored [66,78,79]. Another question is whether the study quantify glucoside forms, aglycones, or both. There are plenty of data on phenolic content in plants and assessments of dietary intake found in literature. However, these values mostly do not represent the total intake of all polyphenols, even not all flavonoids. In 1976, Kühnau estimated daily dietary intake of polyphenols at 1 g [78]. Later, those values were very often questioned as being almost impossible to attain from natural sources. Studies from the 1990s suggested that daily dietary intake is much lower, e.g., (values in mg/day):

- Denmark—26 [80]
- Finland—0–41 [81]
- Greece—15 [82]
- Japan—60–68 [82]
- The Netherlands—33 [82]
- United States—20 [83]

However, those values mostly referred to only three flavonols and two flavones and therefore were underestimated. Later findings shed new light on polyphenolic intake, including flavonoids. A recent study performed by Pozzo and coworkers on 1658 Italian individuals, aged 45–64 years, revealed a mean intake of flavonoids at 320 mg/person/day, with a median of 251 mg/person/day. Food consumption data were based on the semiquantitative food-frequency questionnaire used in the EPIC (European Prospective Investigation into Cancer and Nutrition) and the flavonoid intake was calculated using the latest food composition tables published by the US Department of Agriculture (USDA) and extended by European data. The database contained information on the food content of seven major classes of flavonoids. Flavonoid intake was calculated by multiplying the specific flavonoid content in food servings (expressed as mg aglycone equivalent/100 g food) by the daily consumption of the selected food item (g/day). The study revealed that the main consumed classes of flavonoids were proanthocyanidins and flavan-3-ols with median intakes of 96.1 and 50.4, respectively. The major consumed substances were, among others, polymers of flavon-3-ols, simple and condensed catechins (theaflavins, thearubigins), hesperetin, myricetin, or quercetin. The major sources of flavonoids were fruits, red wine, vegetables, and tea [84].

Recent studies on polyphenols intake found in the literature are based on calculations using US or European databases, among which the Phenol-Explorer database is one of the most important and widely used. It contains information on the content of 502 polyphenol glycosides, esters, and aglycones in 452 foods and may be helpful in the estimation of daily dietary intake of phenolics [85]. However, values based on estimations using databases are inaccurate, as the content of flavonoids and other polyphenols may vary significantly according to place, harvest time or food processing. Therefore, these values are only approximate and may differ significantly from analytical data, which are hard to collect considering the multitude and structure diversity of dietary polyphenols.

A study performed in Poland on 648 students was based on analytical determinations of selected flavonoids and phenolic acids in reconstructed daily food rations using the HPLC method. Moreover, total phenolic content (TPC) was determined using the Folin–Ciocalteu method. The mean intake of phenolics over the 3-year study period was determined to be 245.8 and 172.7 mg/day in women and men, respectively. The major polyphenol, in both the food rations of women and men, was hesperidin, while quercetin was also consumed in significant amounts—32.6 mg/day among women and 35.8 mg/day among men. The study was limited to the intake of seven phenolic compounds; however, TPC values expressed as gallic acid equivalents were much higher and close to 1 g/day [66].

**Figure 1:**
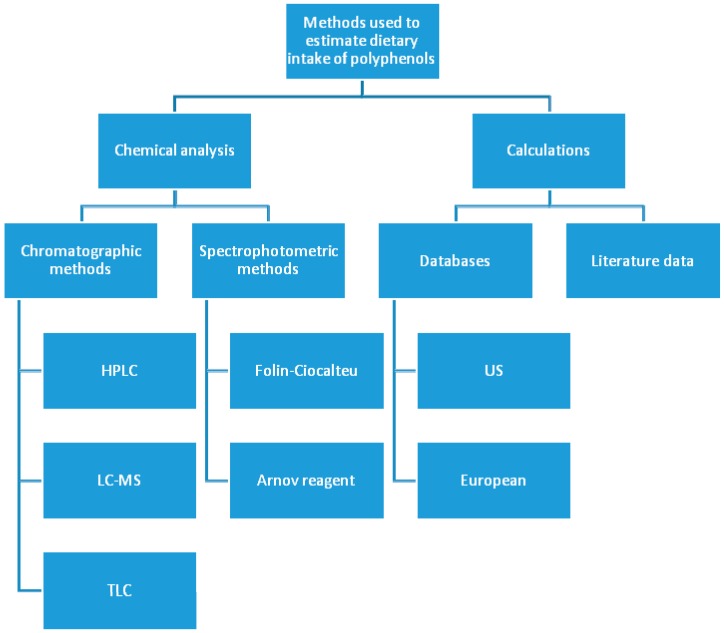
The most common methods used to estimate dietary intake of polyphenols.

According to Pérez-Jiménez and coworkers and based on the Phenol-Explorer database, cloves and dried peppermint are the foodstuffs richest in polyphenols, with content of 15 and 12 g/100 g. Later, food products such as star anise, cocoa powder, Mexican oregano (dried), or celery seed were classified, with contents between 2 and 5 g/100 g [85,86,87]. However, the consumption of these products is so low that they cannot be considered important dietary sources of polyphenols. The Phenol-Explorer database suggests that products rich in phenolics, where the intake may be considered significant (amounts and frequency of consumption), contain between 1756 mg (black chokeberry) to 45 mg/100 g (broccoli) of polyphenols. Although black (102 mg/100 g) and green tea (89 mg/100 g) infusions were classified 52 and 54, respectively, according to the classification of the 100 foods with the highest polyphenol content [87], recently they have been considered the most important sources of dietary polyphenols. The multitude of methods used to evaluate dietary intake of polyphenols caused significant differences between particular results. Figure 1 gathers the most important methods used to estimate the intake of dietary phenolics.

## 4. Dietary Polyphenols—Natural Substances Beneficial in the Prevention and Treatment of Lifestyle Diseases

**Figure 2:**
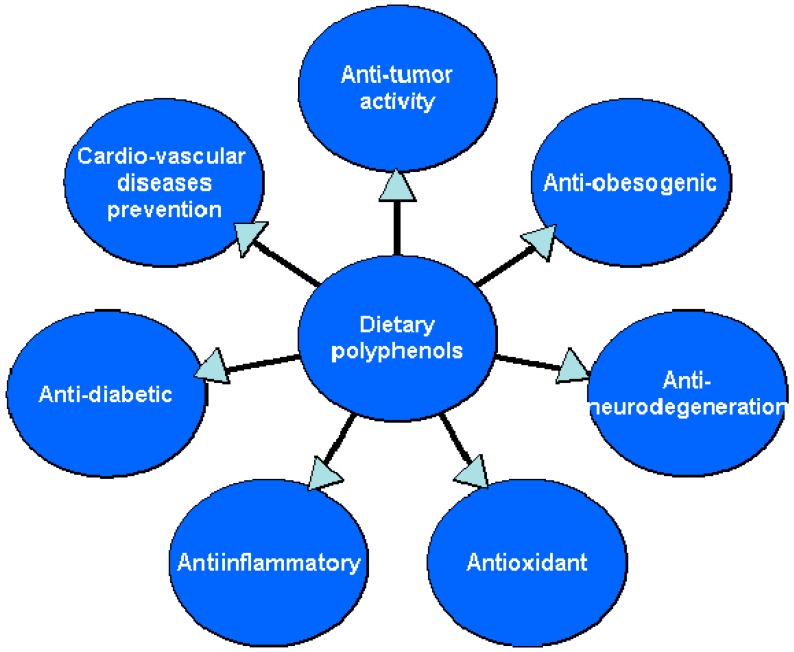
Biological properties of dietary polyphenols important in lifestyle diseases prevention.

A wide spectrum of biological activity of polyphenols has previously been extensively described in numerous scientific papers, including in vitro and in vivo studies. We can see from Figure 2. These natural components of human diet are probably the most extensively described in the scientific literature. Some of their properties may be used in the prevention and treatment of lifestyle diseases. One of the targets of the present review was to gathered some of the interesting and recent findings on the biological properties of selected dietary polyphenols, including molecular mechanism of action, regarding their potential role in reducing the incidence of civilization diseases. Since scientific literature is filled with hundreds of studies focused on biological properties of polyphenols, only research on compounds which dietary intake is significant were considered and described.

### 4.1. Antioxidant Activities of Dietary Polyphenols

This is probably the most extensively and the most frequently described biological activity of phenolics, which because of their structure, may be related to almost all compounds from this group. Experimental studies revealed that the mechanism of action may include several different pathways: direct ROS scavenging; inhibition of enzymes or trace elements chelation, which are involved in free radicals generation (e.g., copper or iron); increase in endogenic antioxidant production (e.g., SOD (SuperOxide Dismutase), glutathione). Flavonoids are known for their properties to inhibit enzymes which are involved in ROS generation, like glutathione S-transferase, microsomal monooxygenase, mitochondrial succinoxidase or NADH oxidase. Protective role, against free radicals, for other antioxidants (e.g., vitamin C and E) was also proved [66,88,89,90,91,92,93]. In vitro experiments showed that quercetin, epicatechin, and rutin are described by strong antioxidant properties. Quercetin possess strong iron-chelating and iron-stabilizing properties. Epicatechin and rutin were proved to be strong ROS scavengers and inhibitors of lipid peroxidation. Moreover, it was shown that glycosidic forms of polyphenols are usually weaker antioxidants than the corresponding aglycones [90]. Green tea polyphenols were confirmed to significantly increase the antioxidant capacity of human blood serum. Clinical study performed on ten healthy Korean subjects (aged 23–25), which consume 150 mL of green tea infusion in the first week, 300 mL in the second, and 450 mL in the third, showed increased total capacity of serum and plasma. The highest changed was observed after drinking of 300 and 450 mL, whereas after ingestion of 150 mL, no significant differences were observed. Moreover, this study elucidated that the highest increase was observed 120 min after tea consumption [94].

Not only simple polyphenols derived from tea, but also the products of their condensation, synthesized during the production of fermented and semifermented teas, possess strong antioxidant properties. Theaflavins, which are one of the most important phenolic constituents present in black tea, were proved to be stronger antioxidants in comparison to EGCG ((-)-epigallocatechin-3-gallate), which is considered one of the most active antiradical molecules present in food. Randomized, double-blind, crossover study performed by Arent and co-investigators revealed that consumption of black tea, enriched in theaflavins, led to better recovery and decreased the oxidative stress in humans subjected to acute anaerobic interval training. It was also observed that theaflavins reduced muscle pain after training in comparison to control group [95]. Imran and coworkers studied the influence of theaflavins and thearubigins, which are a dominant fraction of polymeric polyphenols present in black tea, on antioxidant status and lipid peroxidation in rats. They have proved that theaflavins showed higher antioxidant activity compared to thearubigins. A diet rich in both TFs and TRs (thearubigins) showed significant reduction in lipid profile, glucose content, and renal function. Animals receiving TFs were characterized by lower values in the TBARS (thiobarbituric acid reactive substances) test, compared to thearubigins or a mixture of TFs and TRs (1:1). However, theaflavins+thearubigins diets caused the highest glucose, urea, and creatinine reduction and maximum level of insulin and antioxidant parameters compared to diets containing TFs or TRs alone [96]. Another clinical study showed beneficial effects of oolong tea drinking, which is considered the richest source of another condensed compounds of phenolic nature—theasinensins. Research performed on athletes showed that consumption of oolong tea for 30 days caused significant antioxidant effect observed as strong reduction of malondialdehyde at rest and after exercise, decreased levels of SOD at the rest. It was also observed that oolong tea drinking significantly reduced lipid peroxidation and normalized the cholesterol profiles [97]. Among all thesinensins isomers, theasinensin A was considered as the strongest antioxidant agent against lipid peroxidation [98].

### 4.2. Anti-Inflammatory Effect

Since low-grade, chronic inflammation is a key factor in the development and progression of lifestyle diseases beneficial effects of polyphenols towards inhibition and reduction of any form of inflammation may be crucial in prevention and treatment of this type of disorders. Several studies revealed positive role of tea polyphenols considering inhibition of different mechanisms of inflammation. EGCG was proved to reduce autoimmune response by affecting the differentiation of the dysregulated CD 4(+) T cells to potential autoimmune agents. Animals experiments proved beneficial effects of EGCG treatment towards the improvement of autoimmune diseases [99]. It was also shown that EGCG inhibits proliferation of autoreactive T cells, production of proinflammatory cytokines and Th1 and Th 17 subpopulations, and increases the amount of regulatory T cells populations in lymphoid tissues and the central nervous system. All these findings suggest that EGCG may have preventive and therapeutic potential regarding T-cell mediated autoimmune diseases [100]. Epigallocatechin-3-gallate was also shown to reduce inflammation in connective tissue. EGCG was injected intraperitoneally 3 times/week into mice with collagen-induced arthritis. Significant reduction in cytokines, phosphorylated-signal transducer and activator of transcription-3, 705 and 727 and oxidative stress proteins production was observed. Moreover, the inhibition of osteoclast and Th 17 production was proved associated with increased regulatory T cells production. As a result, significant reduction in arthritis symptoms has been observed [101]. Not only tea catechins, but also other dietary flavonoids were recently described to have immune-response modulation properties. The recruitment of neutrophils to the site of injury or damage is an early sign of inflammation. Significant reduction of NO (nitric oxide), MPO (myeloperoxidase), and TNF-α (inflammatory mediators) production in human peripheral blood neutrophils by rutin, quercetin, and vitexin in the concentration of 25 µM was observed. These result suggests that dietary flavonoids may have a potential role in treating patients with neutrophil-mediated inflammatory diseases [102,103]. Apigenin administrated orally significantly reduced the infiltration of the inflammatory cells in the experimental autoimmune myocardiatitis in mice. The production of proinfalammatory cytokines—Th1 cytokines, TNF-α, interferon-gamma (IFN-γ), and interleukin-2 (IL-2) was also limited [104].

### 4.3. Anticancer Activity

One of the diseases most frequently associated with improper changes in lifestyle, including dietary habits, are different forms of cancer. Among the biological properties of dietary polyphenols, the cancer-chemopreventive properties in various in vitro and animal models have been extensively reported. This activity may originate from their antioxidant and anti-inflammatory properties. Recently, one of the most frequently investigated phenolics towards potential cancer prevention and treatment are simple and condensed tea polyphenols. Zhang and coworkers revealed induction of apoptosis of hepatocellular carcinoma LM6 cells (HCCLM6), associated with the reduction of mitochondrial membrane potential and promotion of G0/G1 phase cell cycle arrest caused by EGCG. The induced apoptosis of HCCLM6 cells was connected with a significant decrease in Bcl-2 and NF-κB expression. No influence on noncancerous liver cells was proved (HL-7702) [105]. Anticancer activity of epigallocatechin-3-galltae was also proved in animal model. Chemopreventive effect of EGCG and TF (theaflavin) in the treatment of tongue and liver cancer induced by N-nitrosodiethylamine in mice was shown. The mechanism of action was associated with the modulation of cellular proliferation/apoptosis and prevalence of CD 44 positive regulation [106]. Significant chemopreventive activity of theaflavins towards prostate cancer was also proved. Two independent studies showed reduction of proliferation and apoptosis induction in human prostate carcinoma (LNaCP and PC-3 cells). Theaflavin caused tumor cell death by induction of p53 suppressor expression and inhibition of nuclear transcription factor NF-κβ and mitogen-activated MAPK-kinase, which are important carcinogenesis factors. The cell cycle blocking in the phase G2/M was also observed [107,108]. Theaflavins are considered as a promising chemopreventive agent in human prostate cancer as their relative bioavailability in prostate tissue was 70% higher in comparison to EGCG [109]. Other dietary flavonoids are also considered as strong, natural anticancer agents. Recently, numerous studies indicated interesting findings on anticancer properties of the most abundant polyphenolic aglycones in the human diet—quercetin, apigenin, and hesperidin. Recently, quercetin was shown to inhibit of the human retinoblastoma (RB Y79 cell line) growth. Inhibition of tumor progression and invasion in the dose dependent manner was proved. The strongest effect was revealed at the quercetin concentration of 100 µM. Additionally antiangiogenesis effect was also reported [110]. Another recent study performed by Hashemzaei and co-investigators showed significant anticancer activity in in vitro and in vivo models. Quercetin used at the concentrations of 10, 20, 40, 80, and 120 µM was found to induce apoptosis of cancer cells of nine different cancer cell lines, i.e., colon, prostate, breast, and ovarian carcinomas. These results were confirmed in animal model, as mice bearing colon and breast cancers exhibited a significant reduction of tumors in groups treated with quercetin [111]. Apigenin, a common dietary flavonoid, present in many fruits and vegetables was recently widely investigated for its anticancer properties. Animal studies showed significant reduction of proliferation, invasion, and migration and increased apoptosis of colonorectal, breast, prostate, melanoma, renal cell, and ovarian cancers [112,113,114,115,116,117,118]. Hesperidin, a flavonoid derived from citrus fruit, was reported to inhibit human hepatic cancer HepG2 cells growth through the activation of caspase-9, -8, and -3, downregulation of Bcl-xL protein, and upregulation of Bax, Bak, and tBid protein levels in a dose-dependent manner. It was interesting that the sensitivity of HepG2 cancer cells to hesperidin was much higher compared to naringin, neohesperidin, and naringenin [119]. Other in vitro experiments, performed on MCF-7 human breast, androgen-independent PC-3, and DU-145 prostate cancer cells, and androgen-dependent LNCa cancer cells, revealed significant antitumor effect of hesperidin. The mechanism of action was different to antimitosis and involved inhibition of the proliferation of MCF-7-GFP-Tubulin cells and both basal and testosterone-induced proliferation of LNCaP cells [120]. Hesperidin was also proved to inhibit ovarian cancer cell viability and increase cytotoxicity in a dose- and time-dependent manner. The mechanism of action involved induction of apoptosis through activation of protein expression of antigrowth arrest- and DNA damage-inducible gene 153, anti-CCAAT’enhancer-binding protein homologous protein, glucose-regulated protein 78, and cytochrome c. This mechanism may shed new light on a potential role of this dietary flavonoid in the prediction of ovarian carcinoma [121].

### 4.4. Cardiovascular Diseases Prevention

A population-based cohort study performed by Pozzo and coworkers over 12 years on 1658 individuals showed that higher dietary intake of flavonoids was associated with reduced risk of CVDs after a mean 12-year follow-up and a 40–50% lower risk of nonfatal CV events. No influence of different subclasses of flavonoids on CV diseases prevention was observed. The intake of flavan-3-ols, anthocyanidins, and flavanones were the most significantly inversely associated with all-cause mortality [84]. Although results from numerous studies and RCTs are inconclusive, a recent meta-analysis performed by Sarrias and coworkers (based on 117 articles published between 1997–2015) revealed significant effect of flavanols on blood lipids. Flavanols from tea, cocoa, and apples (catechins and dimeric procyanidins) were proved to significantly reduce total and LDL cholesterol and increase HDL fraction. No influence on TAGs level was observed [122].

During the last 10 years, several studies and clinical trials have confirmed that catechins have strong antihypertensive activity. The mechanism involves regulation of NADPH production and inhibition of RNA expression of Il-6 and MMP-9 in blood patients with hypertension [123,124,125,126,127,128]. Bogdanski and co-investigators in a double-blind, placebo-controlled trial on 56 obese, hypertensive subjects, revealed that green tea polyphenols are effective in reduction of both systolic and diastolic blood pressures. Additionally, increased intake of these flavonoids caused significant reduction of TNF-α, C-reactive protein levels, fasting serum glucose, insulin levels, and insulin resistance [123].

Rats treated for five weeks with (-)-epicatechin at 2 or 10 mg/kg per day were shown to be resistant to a systolic blood pressure increase caused by administration of deoxycorticosterone acetate (DOCA). Epicatechin was proved to improve the impaired endothelium-dependent relaxation response to acetylcholine and reduce plasma endothelin-1 and malondialdehyde levels [126]. Among other catechins, EGCG was especially responsible for the inhibition of calcium-activated chloride channels. Further, based on that mechanism, calcium dependent phosphorylation of inositol triphosphate, calmodulin antibodies, and calmodulin-dependent protein kinase II were improved, which resulted in strong antihypertensive effect [129,130]. Studies performed on spontaneously hypertensive rats revealed significant decrease of blood pressure after administration of EGCG (200 mg/kg/day). The effect was comparable to observed after treatment with 3 mg/kg/day of enalapril—a common hypotensive drug. Moreover, it was shown that EGCG increased production and release of nitric oxide from endothelium via a PI-3-kinase pathway. Obtained results suggested that EGCG may play beneficial role in the treatment of hypertension and metabolic syndrome [131]. EGCG was also proved to inhibit renin—a crucial enzyme in the treatment of hypertension. This in vitro study also revealed that the specific structure of EGCG—a galloyl moiety and ortho-trihydroxy phenyl structure—may be responsible for the inhibitory activity of EGCG towards renin [132].

Catechins can also play a beneficial role in the prevention of hypertension through the increased release of NO, which is endogenous relaxing factor, responsible, among others, for lowering blood pressure. EC was proved to increase the activity of endogenous NO synthetase, protect endothelial cells from strong stress, and stabilize EDRF (endothelium-derived relaxing factor) when blood vessels are damaged [128].

### 4.5. Anti-Obesity Effect/Weight-Reducing Properties

According to Huang and coworkers, until 2013 there were about 24 different clinical trials performed on obese participants, which have proved the weight-reducing properties of tea polyphenols [133]. Numerous studies performed in vitro and on animals have also confirmed beneficial effects of green tea infusions on weight reduction. The precise molecular mechanism is still discussed. However, the key findings involve reduction of food intake, deterioration of lipid emulsification and absorption, adipogenesis and lipid synthesis suppression, and increase of energy expenditure via thermogenesis. Acceleration of fat oxidation and fecal lipid excretion were also confirmed. As a result, total body mass, fat tissue content, and waist to hip ratio were reduced. Blood lipids levels were also improved [133,134,135,136]. Not only flavan-3-ols present in tea, but also other flavonoids present in commonly consume foods may have a positive effect on the reduction of the body weight. Supplementation with a mixture of quercetin and green tea extract for four weeks in mice fed a high fat diet for 12 weeks revealed significant changes in genes expression. Over 100 genes in adipose tissue were downregulated, including those involved in leukocyte extravasation and phagocytosis. Downregulation of cholesterol metabolism was also observed and cholesterol congeries in adipose and muscle tissue is frequently observed in obesity and sarcopenia. A mild improvement of glucose tolerance in animals fed with quercetin only was proved. This study confirmed other findings suggesting an important role of quercetin, which improved blood glucose tolerance and insulin sensitivity [137,138,139]. Quercetin was also shown to regulate hepatic gene expression related to lipid metabolism in high-fat diet (HFD)-induced obesity in mice. The supplementation with this dietary flavonoid caused significant reduction of liver and white adipose tissue weight as well as hepatic lipid accumulation. Moreover, the size of lipid droplets in the animal epididymal fat pads was also decreased [140].

Luteolin, was also found to increase insulin sensitivity and decrease low-grade chronic inflammation observed in the obesity through the enhanced uptake of glucose by adipocytes and reduction of proinflammatory chemokines blood levels. Moreover, luteolin treatment decreased mRNA levels of tumor necrosis factor α, IL-6, and MCP-1, while it increased the gene expression of adiponectin and leptin in 3T3-L1 adipocytes and primary mouse adipose cells. A potent transcriptional activation of PPARγ (peroxisome proliferator-associated receptor) target genes in animal adipose cells was also revealed [141].

### 4.6. Antidiabetic Activity

Diabetes is the only disease that in the twentieth first century was referred to as an epidemic by the United Nations. It is the most frequently observed chronic noncommunicable disease, which is a major public health problem worldwide. Over 9% of adults have diabetes, which corresponds to 415 million people. Since diabetes is a dominant cause of mortality, morbidity, disability, and increasing healthcare expenditures, this disease is one of the biggest medical challenges of the present time [142]. Several antioxidant, anti-inflammatory, and increasing the insulin sensitivity activities of dietary polyphenols were described above. However, in recent years, many different properties of phenolics towards the regulation of carbohydrates metabolism have been described in the scientific literature. Apigenin, a flavone commonly present in celery, red pepper, parsley, and lemon, was described to have beneficial effects on AMP-dependent regulation of lipid and glucose metabolism. Apigenin was found to be a 200-fold more potent AMPK (5’ adenosine monophosphate-activated protein kinase) activator compared to metformin, a well-known antidiabetes drug. Apigenin administrated intraperitoneally for seven days (4 mg/kg body weight/day) had a strong antihyperglycemic effect in mice with STZ (streptozocin)-induced diabetes. This effect was more marked in the oral glucose tolerance test. The protective effect of these compounds over liver and kidneys of STZ-diabetic models was also shown [143,144,145]. Another study showed that apigenin increased glucose uptake, thyroid hormones level, and insulin secretion in alloxan-induced diabetic mice [146]. Increased level of glucose led to intensified toxicity, which contributes to progressive pancreatic β-cell failure and the development of overt diabetes. Increased production of ROS is one of the major aspects of glucose toxicity. A study performed on HIT-T15 pancreatic β-cells subjected to oxidative stress caused by 2-deoxy-D-ribose (dRib) revealed the protective effect of apigenin against cell damage. The results revealed that this dietary flavonoid caused significant reduction of the intracellular ROS level, increased cell survival and mitochondrial membrane potential, and decreased cell apoptosis. Taken together, it was concluded that administration of apigenin attenuated all the pancreatic β-cell failures caused by dRib [147]. Naringin and naringenin (the aglycone form of naringin), the most abundant representatives of flavanones in foods, possess a wide spectrum of antidiabetic activity. In mice with type 2 diabetes, naringin and hesperidin, which were separately administrated, significantly influenced glycolysis and gluconeogenesis and thus improved hyperglycemia. The proposed mechanism involved the upregulation of hepatic and adipocyte PPARγ and GLUT 4, important for the regulation of glucose metabolism. Additionally, both flavonoids improved lipid metabolism by reducing free fatty acid and TAGs (Triglycerides) plasma levels, inhibition of hepatic (HMG-CoA) reductase activity, and increasing fecal cholesterol [145,148,149]. On the other hand, recent findings on rodents suggest that naringenin and quercetin attenuates glucose uptake in adipose tissue, thus deteriorating glucose metabolism. This was observed after oral administration of these two dietary flavonoids in the amount equal to a consumption of a one glass of grapefruit juice. Glucose tolerance impairment was probably due to inhibition of hypothalamic PI3K signaling, an enzyme which is crucial for the neuronal control of glucose homoeostasis in the organism. These inconclusive actions may be due to individuals’ differences in absorption and metabolism of these polyphenols [150]. Recently, several studies revealed that diet supplementation with hesperidin in db/db, C57BL6 mice resulted in the regulation of hepatic enzymes involved in glucose metabolism in liver, which finally improved hyperglycemia. The reported mechanism was due to the upregulation of hepatic glucokinase, PPARγ, and adipocyte GLUT4. Reduction of G6Pase (glucose-6-phosphatase) and increased expression of GK (glucokinase), which, when combined, decreased glucose export through glucose transporter membrane proteins was observed in rats with STZ-induced type 1 diabetes [148,151,152,153,154]. Quercetin and its glycosylated forms are another example of the flavonoids which are present in human diet in the highest amounts, for which many findings on their antidiabetic activity were recently described. Berry extract with high quercetin concentration was proved to induced AMPK signaling pathways in muscle tissue and oxygen utilization in mitochondria—an analogous mechanism to that of metformin [155]. Quercetin glycosides like hyperoside or isoquercetin also stimulated AMPK in muscle tissue and improved insulin resistance. High effectiveness of quercetin was also reported in the treatment of STZ-induced diabetes in mice. Significant enhancement of liver glucose uptake and reduction of liver glycogenolysis and gluconeogenesis together was proved. Moreover, depletion of GLUT4 and GK stimulated by hyperglycemia was also observed [156,157,158].

Rutin at the concentration of 100 mg/kg in the diet showed significant reduction in blood glucose levels and increased production of insulin in mice with induced diabetes. Moreover, reestablishment of glycogen content and activities of enzymes involved in carbohydrates metabolism was also proved in this study [159].

Several studies proved that supplementation of 0.04–0.045% quercetin in the diet significantly reduced serum glucose and insulin resistance, measured as HOMA-IR parameter, in obese rats and a mouse model. No influence on body weight or adipose tissue size was observed. It was also concluded that quercetin is more effective as an antidiabetic than as an anti-obesity agent [160,161].

Polyphenolic compounds from green, black, and oolong tea were shown to be strong noncompetitive inhibitors of α-glucosidase—the enzyme responsible for intestinal maltose, maltotriose, and α-dextrin hydrolysis, which increases blood glucose levels. Among the tested products, polyphenols derived from oolong tea were characterized by the strongest inhibitory potential [162]. The study performed by Johnston and coworkers on polarized Caco-2 intestinal cells revealed that catechins from green tea are effective inhibitors of glucose intestinal uptake through regulation of the SGLT-1 and GLUT 2 pathways. The authors concluded that such a mechanism may be beneficial in reducing sugar intestinal absorption and an important dietary tool in the management of diabetes [163].

### 4.7. The Activity of Dietary Polyphenols toward CNS Disorders

Neurodegenerative diseases are progressive disorders of the nervous system that are associated with abnormal functioning or destruction of neurons in the brain and/or spinal cord. These diseases are the result of many toxic processes, eventually leading to the death of nerve cells [164]. Factors that may induce neurodegeneration include: deficiency of endogenous antioxidants, inflammation, excitotoxicity of glutamate, expression of proapoptotic proteins, and increases in iron and nitric oxide, leading to oxidative stress [165,166]. Alzheimer’s and Parkinson’s diseases are considered the most common neurodegenerative disorders.

#### 4.7.1. Parkinson’s Disease

PD (Parkinson’s disease) affects about 1% of the population over the age of 50. Symptoms are caused by degenerative changes of dopaminergic neurons, mainly in the substantia nigra pars compacta with the presence of Lewy bodies and Lewy neurites, which results in a decrease in dopamine concentration [167,168]. Other neuropathological changes observed in the disease include: activation of microglial cells and increase in α-synuclein concentration [169]. Despite the many hypotheses and speculations about PD etiology, oxidative stress seems to be a crucial factor in the development of the disease [170]. Although the estimation of daily dietary intake of polyphenols is complicated and their bioavailability and the chemical forms in which they directly act in cells are even harder to explain, recent studies have shown that phenolics can cross the blood–brain barrier and exert biological effects in CNS [171,172,173]. The precise protective mechanism of polyphenols toward the dopaminergic neurons is still unclear and a matter of discussion. Recent studies have confirmed that their major antioxidant mechanism of action is probably not the only one responsible for cellular protection [174]. Fraga suggests that changes in membrane and protein functioning, even at very low polyphenol concentrations, could significantly change molecular mechanisms and therefore possess important biological activity in vivo [175]. Interactions between polyphenols and proteins, even at concentrations lower than those required to act as an antioxidant, leading to inhibition of protein aggregation, play a key role in the prevention of PD. Several studies during the last decade have proved that polyphenols significantly inhibit the expression of α-synuclein—a key protein in the formation of oligomeric aggregates, which is one of the most important factors in the pathogenesis of PD [176,177].

Masuda et al. [178] tested 79 compounds belonging to 12 different chemical classes (including 39 polyphenols) for their potential to inhibit the deposition of α-synuclein into the filaments in vitro. A significant decrease in sarkosyl-insoluble α-synuclein and a corresponding increase in the level of sarkosyl-soluble protein was observed in the presence of exifone, gossypetin, myricetin, and purpurogallin. The most effective polyphenols for the inhibition of α-synuclein filament deposition were baicalein, delphinidin, EGCG, exifone, GC ((-)-gallocatechin), GCG ((-)-gallocatechin gallate), gossypetin, hinokiflavone, hypericin, procyanidin B1, procyanidin B2, rosmarinic acid, and theaflavine. These findings proved that the inhibitory potential of some dietary polyphenols against the formation of insoluble oligomers and reduction of their toxicity to nerve cells may be a crucial mechanism in the prevention of PD development.

In a similar study, Meng et al. [179] investigated the ability of 48 flavonoids belonging to different classes for their effectiveness to inhibit the fibrillation of α-synuclein in vitro. The investigation revealed that the majority of the tested polyphenols inhibit α-synuclein fibrillation and aggregation (e.g., luteolin, rutin, C, ECG (epicatechin-3-gallate)), with EGCG, myricetin, baicalein, eriodictyol, and quercetin being the strongest inhibitors. On the other hand, diosmetin, hesperetin, and hesperidin possessed no inhibitory activity.

Several studies have proved that EGCG can strongly reduce α-synuclein neurocytotoxicity by transforming large, harmful fibrils into smaller, nontoxic, amorphous protein aggregates [180,181,182].

In vitro studies by Lu et al. [183] performed on the rat liver cytosol demonstrated that EGCG is a strong inhibitor of the catechol-O-methyltransferase enzyme (COMT), which can significantly improve the bioavailability of L-Dopa—the most important drug in the therapy of PD.

The protective role of tea polyphenols against neurotoxins has been proved in vivo in mice with MPTP (N-methyl-4-phenyl-1,2,3,6-tetrahydropyridine)-induced Parkinsonism. This study demonstrated that green tea flavonoids, as well as isolated EGCG, strongly decrease striatal dopamine depletion and loss of neurons of the substantia nigra [184]. The neuroprotective role of EGCG was associated with its iron-chelation and antioxidant properties, which led to decreased accumulation of iron and α-synuclein in the substantia nigra pars compacta of the brain [185].

Another study on a mice model revealed that tea consumption decreases, by about 30–60%, the risk of PD. Oral administration of black tea infusions before or after 6-OHDA (a neurotoxin of dopaminergic system applied to rat neonates which develops persistent brain damage) has been reported to reduce the impairment of dopaminergic neurons and improve motor efficiency [186].

Since oxidative stress, accumulation of transition metals, and inflammation play key roles in neuronal damage, the strong antioxidant and metal chelating properties of polyphenols also seem to be crucial in the prevention of neurodegenerative disorders. However, other mechanisms are also significant, as presented above.

Although several in vitro or in vivo experiments have demonstrated the beneficial role of polyphenols against PD, there are still little data from epidemiological studies. One of the few such studies is research performed by Checkoway and co-investigators [187], which proved that consumption of at least two cups of tea per day significantly reduces the risk of PD development. In another study, Gao and coworkers [188] investigated the influence of flavonoid intake on the risk of PD. They concluded that there is a significant negative correlation between a diet rich in flavonoids and PD incidence; however, the protective role of other dietary substances of plant origin also cannot be precluded. They also determined the most important dietary sources of flavonoids in relation to PD, which were berries, red wine, orange/orange juice, and apples (in men), but surprisingly not tea. They also found significant associations between higher anthocyanin intake and PD risk. For other subclasses of flavonoids, such a correlation was not observed.

The pathogenesis of PD is still not clear. Therefore, the future discovery of new findings and mechanisms of action of flavonoids in terms of the prevention of this disease are probably only a matter of time. However, data on the flavonoids dosages necessary to prevent PD incidence and progression and results from clinical studies are currently scarce.

#### 4.7.2. Alzheimer’s Disease

Alzheimer’s disease (AD) is the most common neurodegenerative disorder worldwide and the most frequent observed form of dementia [189]. As reported in 2015, the prevalence of this disease has exceeded 44 million people across the world and it is expected that this number will double by 2050 [190]. The abnormal extracellular accumulation of amyloid-β-peptide in amyloid plaques and hyperphosphorylation of tau proteins and their excessive aggregation in intracellular neurofibrillary tangles are considered to be the most important neuropathological features of AD [191]. Additionally, epidemiological and clinical data support other hypotheses of the disease development: the reduction of the choline acetyltransferase activity [192]; impairment of the brain mitochondria functioning [193]; metabolic changes connected with lifestyle diseases such as diabetes or obesity [194] or vascular problems leading to a deterioration in cerebral blood flow [195].

The positive role of flavonoid-rich products and diets toward the prevention of AD and improving cognitive functions has been extensively described throughout the last 20 years [196,197,198]. In general, their mechanism of action is connected with antioxidant activity and thus anti-aging properties. Protective effects of various polyphenols have been confirmed in several experiments regarding the use of single phenolics, as well as total extracts. Joseph et al. [199] revealed that mice fed for eight months with a diet containing blueberry extract (20 g/kg) rich in polyphenols showed a significant improvement in memory and cognitive functions. Polyphenols from the extract enhanced neuronal signaling via the influence on the extracellular signal-regulated kinase and decreased activity of N-Sase (neutral sphingomyelin-specific phospholipase C) in the hippocampus and striatum. The latter enzyme activity increases as a function of age; thus, its inhibition may have a nootropic effect. The authors concluded that a diet rich in antioxidant phytochemicals may overcome genetic predispositions to AD. However, they did not investigate which particular polyphenols were responsible for such activity.

Of all the dietary polyphenols, it seems that the strongest potential in the prevention of AD has been confirmed for EGCG. This main green tea polyphenol, via activation of PKC (protein kinase C), promotes the nonamyloidogenic α-secretase pathway of APP in neuronal cell cultures and thus reduces the formation of β-amyloid fibrils. These results are supported by in vivo studies—a two-week treatment of mice with EGCG revealed a significant decrease in APP production and an increased synthesis of sAPP (soluble form of APP) in the hippocampus [200,201].

Another clinical parameter associated with a higher prevalence of AD is homocysteine. Increased levels of this amino acid may be responsible for vascular impairment and further neurotoxic alterations [202]. Higher homocysteine levels are determined in the moderate phase of AD, rather than in initial stage and control groups. Recent findings suggest that consumption of beverages rich in polyphenols may reduce inflammation and vascular impairments associated with AD. Antioxidants decrease the levels of thiol groups (from homocysteine), which are involved in the autocatalytic oxidation process in plasma and increased production of ROS. As a result, lower amounts of free radicals and reduced cell damage are observed [203]. A population-based prospective study preformed on 1836 Japanese-Americans demonstrated that consumption of fruit and vegetable juices at least three times per week significantly delayed the onset of AD, especially among patients who are at high risk of developing the disease. In particular, polyphenols from grapes were emphasized as being active toward the reduction of the disease progression [203,204].

Moderate consumption of red wine (rich in stilbenes, ex. resveratrol) has been proved to reduce β-amyloid progression and thus attenuates cognitive impairment and the prevalence of AD [205,206].

Table 2 summarizes recent findings on the biological properties of dietary polyphenols, which may be crucial in the potential prevention of lifestyle diseases incidence.

## 5. Conclusions

Polyphenols are the most common non-nutrients present in the human diet. Considering the significant amounts of compounds consumed in this class and the multitude of their activities, it should be noted that they can play an important role in the prevention of numerous disorders, including civilization diseases. However, it should be remembered that despite many promising results obtained in in vitro or animal experiments regarding their beneficial effects towards the organism, there is still not enough convincing evidence from human studies, especially with large populations. More research of this type is needed to better understand the real value of dietary polyphenols in the context of their ability to prevent the progress of civilization diseases.

## Figures and Tables

**Table 1:** Diet and lifestyle characteristic of hunter-gathers which resembled the habits of Paleolithic societies.

No	Characteristic	References
1	regular sun exposure (except the populations with high intake of vitamin D_3_ from fish and marine mammals, for which endogenous synthesis was less relevant, e.g., the Inuit)	[11,12,13,14]
2	sleep patterns according to natural day/night rhythm	[15]
3	lack of chronic stress/presence of acute stress	[16]
4	regular high physical activity—required to get food, to protect against predators or enemies, to building shelters, and to make social interactions	[17,18,19]
5	low environmental exposure to anthropological pollutants (e.g., pesticides, toxic heavy metals, cancerogenic carbohydrates etc.)	[16]
6	fresh (generally unprocessed) food a. plant leaves, seaweed, sea grasses, and algae b. roots and tubers c. berries and wild fruits d. insects, fish, marine animals, reptiles, and birds e. wild terrestrial mammals and eggs f. nuts and seeds g. honey (occasional intake)	[3,9,10,20,21,22,23]

**Table 2:** Health benefits of dietary polyphenols.

Bioactivity	Experimental Model		Compound	Mechanism of Action/Effect	Ref.
	In Vitro	In Vivo			
Antioxidant	DPPH, ABTS, FRAP assays		Quercetin, rutin, EC,	—direct ROS scavenging —trace elements chelation —increasing the endogenic antioxidant enzymes production	[66,88,89,90,91,92,93]
		Ten healthy Korean subjects	Green tea polyphenols	—increased total capacity of blood plasma and serum	[90]
		People subjected to anaerobic trainning	Theaflavins	—decreasing the oxidative stress in humans subjected to acute anaerobic interval training	[95]
		Rats	Theaflavins and thearubigins	—increasing the antioxidant parameters of blood —significant reduction in lipid profile, glucose content and renal function —increasing the insulin level —the best results after administration of both theaflavins and thearubigins simultaneously	[96]
		Athletes	Theasinensins from oolong tea	—strong reduction of malondialdehyde at rest and after exercise —decreased levels of SOD —reduced lipid peroxidation and cholesterol profile	[97]
Anti-inflammatory	Different immune cells		EGCG	—decreasing the production of proinflammatory chemokines —inhibition of the proliferation of autoreactive T cells —reducing the production of autoimmune agents	[99,100]
		Mice with collagen-induced arthritis	EGCG	—significant reduction in cytokines, phosphorylated-signal transducer and activator of transcription-3, 705 and 727 and oxidative stress proteins production —inhibition of osteoclast and Th 17 production —increased regulatory T cells production	[101]
	Human peripheral blood neutrophils		Rutin, quercetin and vitexin	—significant reduction of NO, MPO and TNF-α (inflammatory mediators) production	[102,103]
		Mice with an experimental autoimmune myocardiatitis	Apigenin	—inhibition of the proinflammatory cytokines production - Th1 cytokines, TNF-α, interferon-gamma (IFN-γ), and interleukin-2 (IL-2)	[104]
Anticancer	hepatocellular carcinoma LM6 cells (HCCLM6)		EGCG	—induction of apoptosis —reduction of mitochondrial membrane potential and promotion of G0/G1 phase cell cycle arrest —significant decrease in Bcl-2 and NF-κB expression.	[105]
		Mice with tongue and liver cancer induced by N-nitrosodiethylamine	Theaflavins and EGCG	—modulation of cellular proliferation/apoptosis and prevalence of CD 44 positive regulation	[106]
	human prostate carcinoma (LNaCP and PC-3 cells)		Theaflavins	—tumor cell death by induction of p53 suppressor expression and inhibition of nuclear transcription factor NF-κβ and mitogen-activated MAPK-kinase, —the cell cycle blocking in the phase G2/M	[107,108]
	human retinoblastoma (RB Y79 cell line)		Quercetin	—inhibition of tumor progression and invasion —angiogenesis inhibition	[110]
	9 different cancer cell lines (i.a. colon, prostate, breast and ovarian carcinomas). These results were confirmed in animal model, as	Mice with induced colon and breast cancers	Quercetin	—cell growth inhibition of all tested cell lines due to the induction of apoptosis —reduction of the tumor volume in a mouse model and increased animal survival	[111]
		Animal models with induced colorectal, breast, prostate, melanoma, renal cell and ovarian cancers	Apigenin	—reduction of proliferation, invasion and migration —increased apoptosis	[112,113,114,115,116,117,118]
	human hepatic cancer HepG2 cells		Hesperidin	—activation of caspase-9, -8, and -3 —downregulation of Bcl-xL protein —upregulation of Bax, Bak, and tBid protein levels	[119]
	MCF-7 human breast, androgen-independent PC-3 and DU-145 prostate cancer cells, and androgen-dependent LNCa cancer cells		Hesperidin	—inhibition of the proliferation of MCF-7-GFP-Tubulin cells and both basal and testosterone-induced proliferation of LNCaP cells	[120]
	A2780 ovarian cancer cell line		Hesperidin	—induction of apoptosis —increasing cleaved caspase-3 protein expression levels —elevating the expression of proteins of antigrowth arrest- and DNA damage-inducible gene 153 —increasing anti-CCAAT’ enhancer-binding protein homologous protein expression —increasing the expression of glucose-regulated protein 78 and cytochrome c	[121]
Prevention of cardiovascular diseases		population-based cohort study on 1658 individuals	different subclasses of dietary flavonoids	—significant reduction of a risk of nonfatal CV events - the reduction of all-cause mortality parameters was the most significantly correlated with the intake of flavan-3-ols, anthocyanidins and flavanones	[84]
		a double-blind, placebo-controlled trial, on 56 obese, hypertensive subjects	Green tea polyphenols	—reduction of both systolic and diastolic blood pressures —significant reduction of TNF-α, C-reactive protein levels, fasting serum glucose, insulin levels and insulin resistance	[123]
		Rats with induced hypertension caused by deoxycorticosterone acetate (DOCA)	EC	—inhibition of blood pressure increase —improving the impaired endothelium-dependent relaxation response to acetylcholine —reduction of plasma endothelin-1 and malondialdehyde levels	[126]
	FRT cells transfected with human TMEM16A		Green tea polyphenols	—strong inhibition of calcium-activated Cl^−^ channels —ECG and EGCG more strongly inhibited Cl− currents than the other polyphenols	[129]
		Rats with induced hypertension	EGCG	—significant decrease of blood pressure —stimulated nitric oxide production from endothelium through a PI-3-kinase pathway	[131]
	human recombinant renin inhibitor screening assay kit		EGCG	—inhibition of renin with IC_50_ value of 44.53 µg	[132]
Anti-obesity effect/weight reducing properties		24 clinical, placebo-controlled trials, performed on obese patients	Green tea polyphenols (mostly 600–900 mg/day, which is equal to 3–4 cups of green tea)	—significant decrease in total abdominal fat are —inhibition of lipid synthesis —reduced serum triglycerides —reduction of food intake —deterioration of lipid emulsification and absorption, —induction of adipogenesis —increase of energy expenditure —Acceleration of fat oxidation —improvement of fecal lipid excretion	[133,134,135,136]
		Mice fed a high fat diet for 12 weeks	Mixture of quercetin and green tea extract administrated for four weeks	—downregulation of over 100 genes in adipose tissue, including those involved in leukocyte extravasation and phagocytosis —downregulation of cholesterol metabolism —reduction of cholesterol congeries in adipose and muscle tissue —mild improvement of glucose tolerance	[138]
		Mice fed a high fat diet for 9 weeks	Diet enriched with quercetin in the concentration of 0.025% (*w*/*w*)	—regulation of hepatic gene expression related to lipid —significant reduction of liver and white adipose tissue weight —decrease in hepatic lipid accumulation —reduction of the size of lipid droplets in the animal epididymal fat pads	[140]
	3T3-L1 cells/isolated mouse epididymal adipose cells from C57BL/6J mice		Luteolin (20 μmol/L)	—insulin sensitivity increase —decrease of low-grade chronic inflammation —improved uptake of glucose by adipocytes —reduction of proinflammatory chemokines —reduction of mRNA levels of tumor necrosis factor α, IL-6 and MCP-1 —increasing the gene expression of adiponectin and leptin —activation of PPARγ target genes in animal adipose cells	[141]
Antidiabetic activity		streptozotocin-induced diabetic Wistar rats	Apigenin/rutin/geinistein/luteolin administrated intraperitoneal-ly for seven days (4 mg/kg body weight/day)	—strong hypoglycemic effect, especially observed in the oral glucose tolerance test —protective effect towards liver and kidneys	[143]
	HepG2 hepatocytes	type 1 diabetic LDL receptor-deficient mice (DMLDLR(-/-)	Apigenin <0.1% in DMSO (in vitro) 130 mg/kg/day (in vivo) for six weeks	—regulation of lipid and glucose metabolism through AMP-dependent kinase —prevention of lipid accumulation in the liver —inhibition of hyperlipidemia —slowing the aortic lesion development	[144]
	HIT-T15 pancreatic β-cells		Apigenin (20 mM in DMSO)	—inhibition of pancreatic β-cells damage through different oxidative stress related signaling pathways (decreasing ROS production, apoptosis and increasing the mitochondrial membrane potential)	[147]
		Alloxan-treated diabetic mice	Apigenin (0.78 mg/kg/day in DMSO), s.c. for ten consecutive days	—increased serum insulin and thyroid hormone levels —reduction of blood glucose —reduction of hepaticG-6-Pase activity —reduction of serum cholesterol level —hepatoprotective activity (observed as decreased levels of lipid peroxidation and increased levels of catalase, SOD or glutathione)	[146]
		Mice fed with high-fat diet	Naringin (0.2 g/kg diet, administrated orally)	—inhibition of inflammation and insulin resistance by blocking activation of the MAPKs pathways and by activating IRS-1 —lipid reduction by synthesis inhibition and increased fatty acid oxidation —hypoglycemic effect through the regulation of PEPCK and G6pase activity in the liver	[149]
		Male diabetic C57BL/KsJ-db/db mice	Naringin and hesperidin (0.2 g/kg diet, administrated orally, separately)	—increasing the glucokinase mRNA level (both flavonoids) —reducing the mRNA expression of PEPCK and G6pase in the liver (naringin) —reducing the expression of hepatic glucose transporter 2 protein in the liver —upregulation of the expression of adipocyte GLUT-4 and hepatic and adipocyte PPARγ (both flavonoids) —reducing free fatty acid and TAGs plasma levels (both flavonoids) —inhibition of hepatic (HMG-CoA) reductase activity —increasing fecal cholesterol	[148]
		Adult Djungarian hamsters/ Female C57BL/6JRj-mic	Naringenin and quercetin (10 mg/kg administered orally)	—impaired glucose tolerane —no influence on circulating levels of insulin and insulin-like growth factor-binding proteins —reduced respiratory quotient (impaired glucose utilization) (oral quercetin)	[150]
		Rats with streptozotocin (STZ)-induced marginal type 1 diabetes	Hesperidin (10 g/kg diet)	—reduced blood glucose level by decreased activity of G6Pase and increased expression of GK —decreased glucose export through glucose transporter membrane proteins	[151]
		Male C57BL/KsJ-db/db mice	Diet supplemented with hesperidin (0.2 g/kg diet) or naringin (0.2 g/kg diet)	—significant reduction of blood glucose (both flavonoids) —significant increase of GK activity and glycogen concentration (both flavonoids) —reduced activity of hepatic G6pase and PEPCK (naringin) —increased levels of Plasma insulin, C-peptide, and leptin (both flavonoids)	[152]
		Male Wistar diabetic rats	Hesperidin (100 mg/kg/day) administrated orally	—significant mean arterial pressure improvement —reduced left ventricular end-diastolic pressure —improved both inotropic and lusitropic function of the heart —decreased level of TBARs —anti-apoptotic and protective effect	[154]
	C2C12 murine skeletal myoblasts and H4IIE murine hepatocytes		Quercetin and its glycosylated forms isolated from *Vaccinium vitis-idaea* extract	—significant glucose uptake through the insulin-independent AMPK pathway —mild inhibition of mitochondrial ADP-stimulated oxygen consumption —inhibition of ATP synthase in mitochondria (only the aglycone)	[155]
		Alloxan induced Swiss albino mice	Oral supplementation with quercetin (20 mg/kg body weight) for three weeks	—significant decrease of fasting blood glucose level —reduced markers of liver and kidneys injury —increased levels of antioxidant enzymes (SOD, glutathione, catalase and glutathione-S-transferase) —decreased concentration of TBARs —increased level of GLUT4 expression	[156]
		STZ-induced diabetic mice	Quercetin administrated orally (0.1 and 0.5% of the diet)	—decreased blood glucose —increased insulin plasma level —suppression of the STZ-induced alteration of gene expression (0.5% of quercetin) —the strongest inhibitory effect towards elevated expression of cyclin-dependent kinase inhibitor p21(WAF1/Cip1) (Cdkn1a)	[157]
	3T3-L1 adipocyte cells		Quercetin	—inhibition of insulin-mediated GLUT4 translocation —improved glucose metabolism through the regulation AMPK activity —improvement of insulin cell sensitivity by inhibition of IκB kinase β (IKKβ) phosphorylation and insulin signaling facilitation	[158]
		Diabetic mice	Quercetin administrated orally (0.04 and 0.08% of the diet) for six weeks	—dose dependent decrease of blood level glucose —decreased values of the HOMA-IR parameter —increased plasma adiponectin level (0.08% of quercetin) —reduced levels of LDL, total cholesterol, TAGs and increased HDL —decreased TBARs —increased concentration of the liver SOD, catalase and glutathione peroxidase	[160]
		Rats fed with high sucrose diet	Oral supplementation with quercetin (30 mg/kg body weight per day) for six weeks	—no reduction on body weight or adipose tissue size was observed —reduced levels of fructosamine, basal glucose, insulin, and HOMA-IR —no influence on lipogenic enzymes and lipoprotein lipase activity was noticed —no change in muscle TAGs content was observed	[161]
		STZ-induced diabetic rats	Rutin administrated orally (100 mg/kg body weight) for 45 days	—decrease of plasma glucose concentration —increased blood insulin level —restoration of glycogen content —protective effect towards pancreatic cells (reduced fatty infiltrate of the Langerhans islets)	[159]
	α-glucosidase inhibition assay		Green, black and oolong tea extracts	—dose dependent inhibitory potential towards α-glucosidase —the strongest activity was revealed for oolong tea extract, followed by black and green tea —the activity of particular extract was attributed to the content of polyphenols	[162]
	polarized Caco-2 intestinal cells		Green tea catechins	—inhibition of intestinal glucose uptake —nonglycosylated dietary polyphenols were the most effective in reduction of glucose uptake under both sodium-dependent and sodium-free conditions	[163]
Protective activity towards Parkinson’s Disease	Human α-synuclein assembly inhibitory test		Several dietary polyphenols (e.g., Tea catechins, apigenin, quercetin, rutin)	—dietary polyphenols are a major class of compounds active towards the inhibition of α-synuclein —tea catechins were characterized by the highest inhibitory activity —common dietary polyphenols (rutin, quercetin or naringenin) were less active	[178]
	α-synuclein fibrillation inhibitory assay		48 flavonoids dissolved in DMSO at concentration of 5, 10, and 20 mM	—majority of flavonoids inhibited the fibrillation of α-synuclein (EGCG, rutin, luteolin) —the inhibition of α-synuclein fibrillogenesis was due to the inhibition of nucleus formation and the inhibition of fibril elongation —structure-activity dependency was noticed (a vicinal dihydroxyphenyl moiety was crucial for the inhibitory activity) —EGCG, myricetin, baicalein, eriodictyol and quercetin were the strongest inhibitors —diosmetin, hesperetin or hesperidin possessed no inhibitory activity —diosmetin, hesperetin or hesperidin possessed no inhibitory activity	[179]
	α-synuclein fibrillation inhibitory assay		EGCG (50 µM)	—EGCG directly converted fibrillar species into benign protein aggregates —inhibition of α-synuclein and amyloid-β fibrillogenesis by EGCG through a direct binding to β-sheet-rich aggregates was proofed —EGCG did not reverse the process of fibril formation —no increase in toxic intermediates formation was noticed	[182]
	Rat liver cytosol		Green tea catechins (EGCG and EGC)	—significant inhibition of catechol-O-methyltransferase enzyme (COMT) —dose-dependent methylation of EGCG by COMT was proofed —EGCG inhibited methylation of L-DOPA, a common drug used in the therapy of PD, and improved its bioavailability	[183]
		Mice with MPTP induced Parkinsonism	Green tea extract and EGCG (0.5 and 1 mg/kg i.p.)	—increased activity of striatal antioxidant enzymes (SOD and catalase) —significant decrease loss of neurons and increase production of dopamine in *substantia nigra* —protective mechanism was proofed to be other than inhibition of MPTP transformation to its active metabolite —the protective effect of green tea polyphenols was identified with brain penetrating activity of polyphenols, its antioxidant activity and iron chelating properties	[184]
		Male C57-BL Mice with MPTP induced Parkinsonism	EGCG (2mg/kg/day, per os, for 10 days)	—decreased levels of striatal tyrosine hydroxylase protein and increased activity of this enzyme —increased in PKCα protein expression inside the striatal dopaminergic fibers —neuroprotective activity through iron-chelating and antioxidant action	[185]
		Rats with 6-OHDA-induced PD	Black tea extract 1.5% (administrated orally before and after 6-OHDA injection)	—a significant neuroprotective and neurorescue effects were observed (increased nigral gluthathione and striatal SOD catalase activity and decreased lipid peroxidation level) —improved spontaneous locomotor activity and dopamine (DA)-D2 receptor binding, —increased striatal DA and 3-4 dihydroxy phenyl acetic acid (DOPAC) level —significant increase of antiapoptotic and proapoptotic protein level —higher improvement in motor and neurochemical deficiency was more visible in rats receiving polyphenols before 6-OHDA administration	[186]
		A case-control study conducted in western Washington State in 1992–2000 on 557 individuals	Tea (black or green)	—significant reduced risk of PD development in population drinking 2 cups/day or more of tea	[187]
		Health Professional Follow-up Study performed on 49,281 men and the Nurses’ Health Study performed on 80,336 women	5 classes of flavonoids (intake based on calculations using database and food frequency questionnaire)	—significant negative correlation between flavonoid intake and PD incidence —the strongest correlation between flavonoids intake and PD risk was found for anthocyanins —the significance of the obtained results was more visible in men, compared to women	[188]
Protective activity towards Alzheimer’s Disease		A cohort study on 1367 subjects above 65 years of age	Dietary flavonoid intake based on calculations	—significant inverse correlation between flavonoid intake and risk of dementia	[196]
		Transgenic mice with genetically induced AD	Diet supplemented with 2% of water blue berry extract	—significant improvement in memory and cognitive functions —improved neuronal signaling through enhanced extracellular signal-regulated kinase and decreased activity of N-Sase in the hippocampus and striatum —diet rich in antioxidant from blue berry extract may overpass genetic predispositions to AD —no influence of particular extract constituents were investigated	[199]
	Human SH-SY5Y neuroblastoma cells/Rat pheochromocytoma PC12 cells	Male C57/BL mice	EGCG (2 mg/kg, per os, for 14 days) (in vivo)	—activation of activates PKCα and PKCε in the membrane and cytosolic fractions of mice hippocampus, which lead to increased production of neuroprotective, nonamyloidogenic sAPPα —reduction of cellular APP holoprotein production, which further reduces the progress of amyloidogenic failure —protective effect against β-amyloid-induced neurotoxicity	[200]
	Fresh, nonaggregated β-amyloid fractions		Wine derived polyphenols (myricetin, morin, quercetin, kaempferol, catechin, epicatechin)	—dose-dependent inhibition of β-amyloid formation —dose-dependent destabilization of preformed β-amyloid aggregation —the activity of particular polyphenols was varied in order: myricetin = morin = quercetin > kaempferol > catechin = epicatechin —the effective concentration was within the range of 0.1–1 µM	[201]
		A cohort, population-based prospective study of 1836 Japanese Americans in King County, Washington	Fruit and vegetable juices, containing a high concentration of polyphenols (>3 times per week)	—significant reduction of the hazard ratio for probable Alzheimer’s disease, especially for population drinking more than 3 times per week —the strongest inverse association was revealed for people with an apolipoprotein Eε-4 allele and those who were not physically active	[204]
		Tg2576 mice (model AD-type amyloid beta-protein neuropathology)	Red wine (Cabernet Sauvignon) diluted in drinking water	—improved cognitive functions —attenuation of β-amyloid deposition in neocortex and hippocampus —increased α-secretase activity —significant activity of red wine towards the inhibition of AD development was associated with its polyphenolic composition	[205]

AD—Alzheimer’s disease; AMPK—5’ adenosine monophosphate-activated protein kinase; APP—amyloid precursor protein; EGC—epigallocatechin; PEPCK—Phosphoenolpyruvate carboxykinase; PPARγ—peroxisome proliferator-associated receptor; ROS—reactive oxygen species; TBARS—thiobarbituric acid reactive substances; SOD—SuperOxide Dismutase

## List of Abbreviations

AD	Alzheimer’s disease
AMPK	5’ adenosine monophosphate-activated protein kinase
APP	amyloid precursor protein
b.w.	body weight
C	(+)-catechin
CNS	central nervous system
ECG	epicatechin-3-gallate
EDRF	endothelium-derived relaxing factor
EGC	epigallocatechin
EGCG	(-)-epigallocatechin-3-gallate
G6Pase	glucose-6-phosphatase
GC	(-)-gallocatechin
GCG	(-)-gallocatechin gallate
GK	glucokinase
GLUT 2 and 4	glucose transporters type 2 and 4
HMG-CoA	3-hydroxy-3-methylglutaryl-coenzyme
IRS-1	insulin receptor substrate 1
MPO	myeloperoxidase
MPTP	N-methyl-4-phenyl-1,2,3,6-tetrahydropyridine
NO	nitric oxide
N-Sase	neutral sphingomyelin-specific phospholipase C
6-OHDA	6-hydroxyydopamine
PD	Parkinson’s disease
PEPCK	Phosphoenolpyruvate carboxykinase
PI3K	phosphoinositide-3-kinase
PKC	protein kinase C
PPARγ	peroxisome proliferator-associated receptor
RCTs	randomized controlled trials
ROS	reactive oxygen species
sAPP	soluble form of APP
SGLT 1	sodium-glucose co-transporter type 1
SOD	SuperOxide Dismutase
STZ	streptozocin
TAGs	Triglycerides
TBARS	thiobarbituric acid reactive substances
TF	theaflavin
TFs	theaflavins
TRs	thearubigins
